# Mortality prediction of the frailty syndrome in patients with severe mitral regurgitation

**DOI:** 10.1007/s00380-022-02184-y

**Published:** 2022-10-17

**Authors:** Jasmin Shamekhi, Baravan Al-Kassou, Marcel Weber, Philip Roger Goody, Sebastian Zimmer, Jana Germeroth, Jana Gillrath, Katharina Feldmann, Luisa Lohde, Alexander Sedaghat, Georg Nickenig, Jan-Malte Sinning

**Affiliations:** Heart Center, Department of Medicine II, University Hospital Bonn, Venusberg-Campus 1, 53127 Bonn, Germany

**Keywords:** Mitral valve regurgitation, Frailty assessment, Frailty, MVR, Geriatric assessment, Nutritional status

## Abstract

In this prospective observational study, we investigated the impact of geriatric syndromes and frailty on mortality and evaluated the prognostic value of different frailty, nutritional, and geriatric assessment tools in high-risk patients with severe mitral valve regurgitation (MR) who were evaluated for mitral valve therapies including surgical, interventional, and conservative treatment options. We prospectively assessed multiple parameters including the CONUT Score, the Katz Index of independence in activities of daily living (ADL), the Fried Frailty Phenotype (FFP), and the Essential Frailty Toolset (EFT) Score in 127 patients with severe symptomatic MR requiring surgical/interventional treatment versus conservative monitoring. We compared their predictive value on mortality including multivariate regression analysis to identify the most suitable tool to predict outcomes in these patient groups. The frailty syndrome as assessed with the CONUT Score, Katz Index, EFT Score, and FFP was associated with higher rates of comorbidities, significantly higher risk scores such as logistic EuroSCORE, EuroSCORE II, and STS-PROM, and significantly higher mortality rates. The EFT Score and FFP were independent predictors of one-year all-cause mortality in our study cohort (EFT Score: HR 1.9, 95% CI 1.2 to 3.2; *p* = 0.01; FFP: HR 1.8, 95% CI 1.1 to 3.1; *p* = 0.015). Geriatric syndromes and frailty are associated with increased mortality in high-risk patients with symptomatic severe MR. The EFT Score and the FFP were independent predictors of one-year all-cause mortality.

## Introduction

Heart valve diseases are widespread throughout the Queryentire population. Apart from aortic valve stenosis, mitral valve regurgitation (MR) is the most common valve disease requiring interventional or surgical therapy. In particular, older adults are frequently affected and the prevalence is thought to be increasing due to the aging population, suggesting the need for better awareness and more accurate diagnostic screening methods.

Predominately older adults with heart valve disease have a high likelihood of presenting with multiple comorbidities, geriatric syndromes, and socio-economic problems [1]. These conditions are associated with immobility, multimorbidity, and frailty [2], leading to disability, hospitalization, institutionalization, and death [1, 2]. In patients with severe aortic stenosis undergoing transcatheter aortic valve replacement (TAVR), frailty has already been shown to have an adverse effect on the outcome and to predict mortality [3–6].

However, it remains a matter of debate whether geriatric syndromes and frailty are associated with mortality in patients with severe mitral valve regurgitation and—even more importantly—which geriatric assessment tool is most suitable to predict outcome in this patient population.

We investigated the effect of geriatric syndromes and frailty on mortality and evaluated the prognostic value of different assessment tools on outcome in high-risk patients with severe mitral valve regurgitation requiring surgical/interventional treatment versus conservative monitoring in a prospective observational study.

## Methods

### Patient population

Between January 2018 and May 2019, 127 consecutive high-risk patients with severe symptomatic mitral valve regurgitation were evaluated for interventional/surgical valve repair or conservative treatment at the Heart Center Bonn. All patients were discussed within the local institutional Heart Team and participated in this study after written informed consent was obtained. The study was approved by the local ethics committee and was conducted in accordance with the 1964 Declaration of Helsinki.

All patients underwent a careful standardized evaluation including laboratory tests, transthoracic and transesophageal 3D echocardiography, coronary angiography, pulmonary function test, angiological examination and, if required, a cardiac CT scan to assess the eligibility for transcatheter valve repair or replacement procedures. In addition, we performed a detailed geriatric and frailty assessment including a series of physical and functional performance test and laboratory examinations summarized in four assessment tools: the CONUT (Controlling Nutritional Status) Score [7], the Katz Index of independence in activities of daily living (ADL) [8], the Fried Frailty Phenotype (FFP) [9, 10], and the Essential Frailty Toolset (EFT) Score [3]. The four geriatric assessment tools are described in detail in the literature [3, 7–10]. Briefly, the CONUT Score includes the assessment of three laboratory parameters: serum albumin, cholesterol, and lymphocytes and reflects the nutritional status of the patient. The KATZ Index is a questionnaire with six items related to independence in activities of daily living, while the FFP combines functional performance tests, such as evaluation of grip strength and walking time, with physical examinations including the level of physical activity, exhaustion in daily life, and weight loss. The EFT Score combines functional (five chair rise test), cognitive (dichotomous variable), and laboratory parameters (hemoglobin and serum albumin). A CONUT Score cut-off of > 2 [11], an EFT Score cut-off of ≥ 3 [12], an FFP cut-off of ≥ 3 [9], and a Katz Index < 6 [8] were considered as a marker of frailty and advanced geriatric syndromes. For the physical health evaluation, we used the body-fat scale OMRON BF511 and assessed the percentage of body fat, visceral fat, and muscle mass, as well as caloric expenditure.

Out of 127 study patients, 100 underwent mitral valve repair (MVR) during the follow-up period. To address a possible selection and therapy bias in our study, we performed a subanalysis and stratified patients according to the different treatment strategies (MVR vs. conservative treatment) and again compared the rates of one-year all-cause mortality.

### Study endpoint, data collection, and follow-up

The primary endpoint of our study was one-year all-cause mortality. The endpoint was predefined prior to the start of the study.

After discharge, clinical follow-up data were prospectively collected during scheduled outpatient clinic visits or direct telephone interviews with the referring cardiologists, general practitioners, and patients.

### Statistical analysis

Data are presented as mean ± standard deviation, if normally distributed, or as the median and interquartile range (IQR) (quartile 1/quartile 3), if not normally distributed. Continuous variables were tested for having a normal distribution with the use of the Kolmogorov–Smirnov test. Categorical variables are given as frequencies and percentages. For continuous variables, a student’s *t*-test or a Mann–Whitney *U* test, as appropriate, was performed for comparing two groups. When comparing more than two groups, ANOVA or the Kruskal–Wallis test was used. Spearman’s correlation coefficients were used to establish associations. The *χ*^2^ test was used for the analysis of categorical variables.

For the statistical analysis, we stratified patients according to their geriatric status and compared baseline characteristics as well as the clinical outcome. Finally, we performed a multivariate regression analysis and a receiver operating characteristics (ROC) curve analysis to identify the most suitable geriatric assessment tool to predict the outcome. Survival according to the different assessment tools was determined using the Kaplan–Meier method. The log-rank test was used to determine statistical differences in terms of survival.

Statistical significance was assumed when the null hypothesis could be rejected at *p* < 0.05. Statistical analyses were conducted with PASW Statistics version 22.0.0.0 (IBM Corporation, Somers, NY, USA) and MedCalc version 11.6.1.0 (MedCalc Software, Mariakerke, Belgium). The investigators initiated the study, had full access to the data, and wrote the manuscript. All authors vouch for the data and its analysis.

## Results

**Fig. 1:**
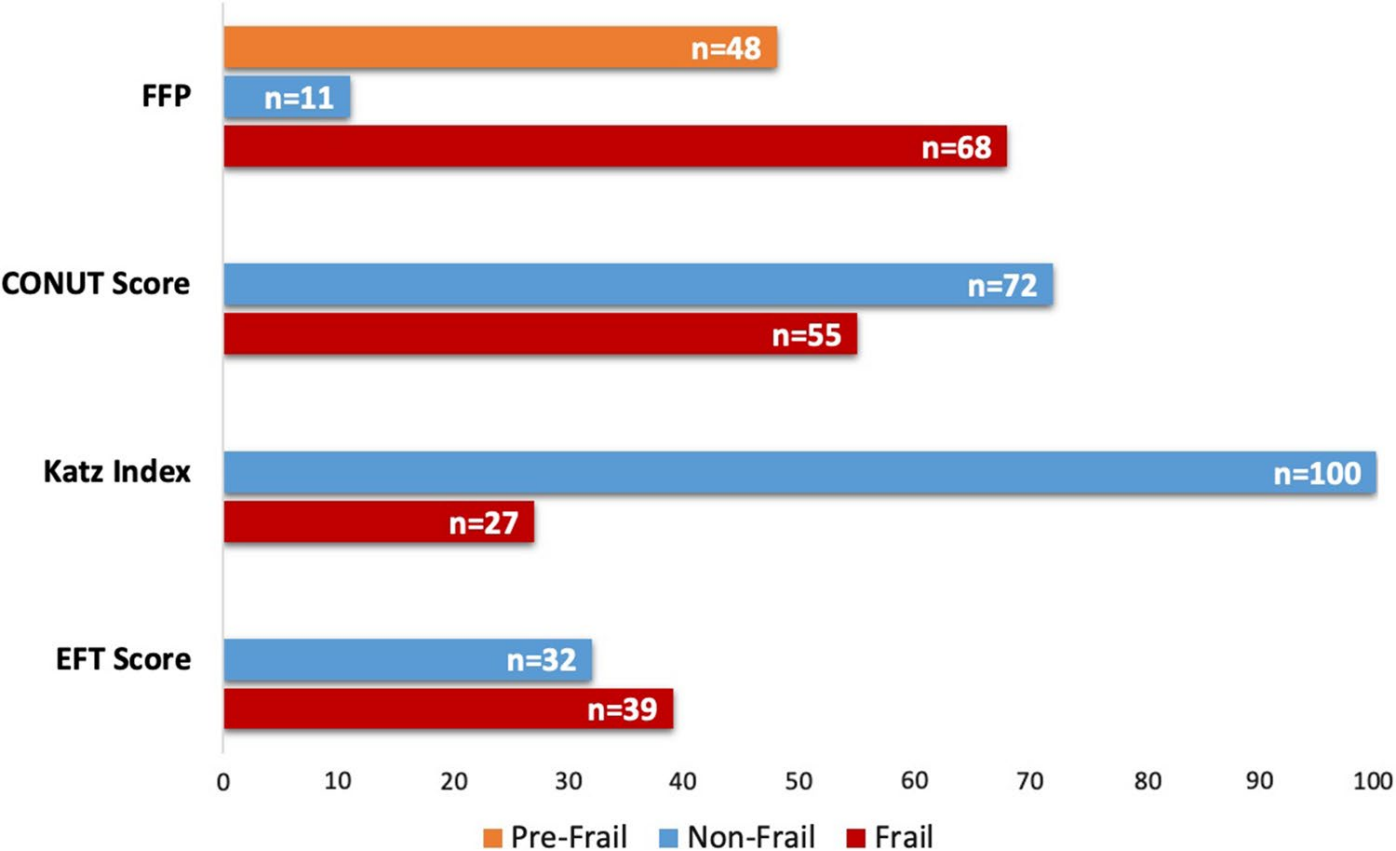
Overall prevalence of geriatric syndromes according to the four scales. The prevalence of geriatric syndromes in patients with severe MR or TR varies depending on the used assessment tool and ranges from 52% assessed with the FFP to 21% using the Katz Index in our study. *MR* mitral valve regurgitation; *TR* tricuspid valve regurgitation; *FFP* Fried Frailty Phenotype; *CONUT Score* Controlling Nutritional Status Score; *EFT* Score Essential Frailty Toolset Score

Baseline characteristics of all patients are presented in Table 1. The mean age of our study population was 75 (± 9.4) years and 49.6% were female. Out of the total of 127 patients, 18 (13.9%) suffered from concomitant moderate or severe TR, 68 (55.3%) patients had a history of coronary artery disease, and the median NT-pro BNP level was 2316 pg/ml (898/5518) in the overall population (Table 1).The overall prevalence of geriatric syndromes in our study cohort according to the different assessment tools is presented in Fig. 1.

**Table 1:** Baseline characteristics of all patients

	All patients, *n* = 127
Age, years	75.7 (9.4)
Female sex, *n* (%)	63 (49.6)
BMI, kg/m^2^	26.6 (5.1)
Creatinine, mg/dl	1.2 (0.97/1.79)
Troponin T, ng/l	23.6 (13.8/42.4)
NT-proBNP, pg/ml	2316.0 (898/5518)
CRP, mg/l	4.5 (1.5/11.6)
Hemoglobin, g/dl	12.2 (10.5/14.0)
Albumin, g/l	40.8 (6.3)
Ejection fraction, %	51.2 (14.3)
EuroSCORE	14.6 (8.9/25.6)
EuroSCORE II	4.5 (2.6/7.5)
STS Prom	3.0 (1.6/4.6)
Extracardiac arteriopathy, *n* (%)	45 (35.4)
Carotid disease, *n* (%)	26 (20.5)
COPD, *n* (%)	27 (21.3)
Hypertension, *n* (%)	99 (78.0)
Diabetes, *n* (%)	35 (27.6)
CAD, *n* (%)	68 (55.3)
Previous cardiac surgery, *n* (%)	29 (22.8)
Atrial fibrillation, *n* (%)	90 (70.9)
TR ≥ moderate, *n* (%)	18 (13.9)

Approximately, normal distributed continuous parameters were compared with Student’s *t*-test, otherwise Mann–Whitney *U* test and *χ*^2^ test were used

*BMI* body mass index, *NT-proBNP* B-type natriuretic peptide, *CRP* C-reactive protein, *COPD* chronic obstructive pulmonary disease, *CAD* coronary artery disease, *MR* mitral regurgitation, *TR* tricuspid regurgitation

### Baseline characteristics according to the frailty scales

**Fig. 2:**
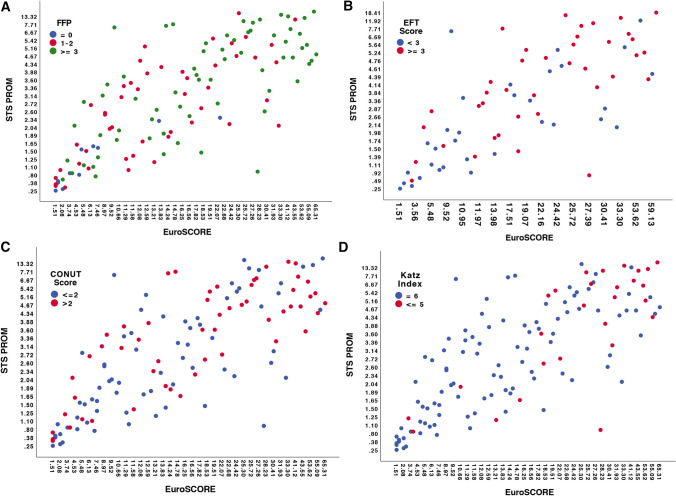
Scatter plots according to risk and geriatric scores. Geriatric syndromes as assessed with the CONUT Score (2C), Katz Index (2D), EFT Sore (2B), and FFP (2A) were associated with higher rates of comorbidities and significantly higher risk scores such as logistic EuroSCORE (lES) and STS-PROM. *FFP* fried frailty phenotype; *CONUT Score* Controlling Nutritional Status Score; *EFT Score* Essential Frailty Toolset Score

Baseline characteristics according to the different geriatric assessment scales are presented in detail in Table 2. Geriatric syndromes and frailty as assessed with the CONUT Score, Katz Index, EFT Sore, and FFP were associated with higher rates of comorbidities and significantly higher risk scores such as logistic EuroSCORE (lES) and STS-PROM, as presented in Fig. 2A–D. In addition, patients with advanced geriatric syndromes also displayed higher troponin T and NT-pro BNP levels as well as higher C-reactive protein and lower hemoglobin values.

**Table 2:** Baseline characteristics according to the four geriatric assessment scales

Baseline characteristics according to the four geriatric assessment scales—Part 1						
	Katz Index		*p* value	CONUT Score		*p* value
	= 6 *n* = 100	≤ 5 *n* = 27		≤ 2 *n* = 72	> 2 *n* = 55	
Age, years	75.3 (9.6)	77.5 (8.1)	0.24	76.6 (9.2)	74.6 (9.5)	0.98
Female sex, *n* (%)	49 (49.0)	14 (51.9)	0.79	45 (62.5)	18 (32.7)	0.001
BMI, kg/m^2^	26.6 (5.2)	26.3 (4.9)	0.94	26.5 (5.3)	26.6 (4.9)	0.52
Creatinine, mg/dl	1.16 (0.9/1.6)	1.65 (1.1/2.2)	0.95	1.1 (0.9/1.4)	1.5 (1.0/2.3)	<0.001
Troponin T, ng/l	20.7 (11.3/33.4)	45.9 (22.8/95.5)	0.20	18.7 (10.5/26.3)	40.4 (19.6/75.5)	<0.001
NT-proBNP, pg/ml	1676 (755/4451)	4096 (2316/16948)	0.003	1411 (605/2851)	4887 (1663/16888)	<0.001
CRP, mg/l	3.5 (1.4/9.1)	8.9 (3.0/28.7)	0.08	2.8 (1.2/8.3)	6.8 (2.6/19.9)	0.015
Hemoglobin, g/dl	12.8 (11.2/14)	10.2 (8.8/11.7)	<0.001	13.2 (11.6/14.2)	10.7 (9.1/12.4)	<0.001
Ejection fraction, %	52.6 (13.9)	46.0 (15.1)	0.036	54.4 (13.6)	47 (14.3)	0.004
sPAP, mmHg	41.0 (17.1)	46.2 (23.0)	0.23	40.6 (18.5)	44.0 (18.6)	0.19
EuroSCORE	13.2 (7.4/22.1)	27.3 (18.3/41.3)	<0.001	13.2 (7.5/23.2)	18.5 (10.9/30.7)	0.023
EuroSCORE II	4.1 (2.1/6.6)	8.4 (5.5/13.7)	<0.001	4.4 (2.3/6.6)	5.4 (3.4/8.9)	0.032
STS-PROM	2.6 (1.5/4.4)	5.2 (2.6/6.9)	<0.001	2.3 (1.4/4.2)	3.7 (2.1/5.2)	0.146
Extracardiac arteriopathy, *n* (%)	31 (31.0)	14 (51.9)	0.044	26 (36.1)	19 (34.5)	0.85
Carotid disease, *n* (%)	19 (19.0)	7 (25.9)	0.43	16 (22.2)	10 (18.2)	0.56
COPD, *n* (%)	18 (18.0)	9 (33.3)	0.084	13 (18.1)	14 (25.5)	0.31
Smoker, *n* (%)	22 (22.0)	10 (37.0)	0.062	17 (23.6)	16 (29.0)	0.42
Hypertension, *n* (%)	78 (78.0)	21 (77.8)	0.98	54 (75.0)	45 (81.8)	0.36
Diabetes, *n* (%)	20 (20.0)	15 (55.6)	< 0.001	17 (23.6)	18 (32.7)	0.25
CAD, *n* (%)	50 (50.0)	18 (66.7)	0.12	37 (51.4)	31 (56.4)	0.57
Previous cardiac surgery, *n* (%)	18 (18.0)	11 (40.7)	0.012	12 (16.7)	17 (30.9)	0.058
Atrial fibrillation, *n* (%)	69 (69.0)	21 (77.8)	0.37	48 (66.7)	42 (76.4)	0.23
Body fat, %	25.0 (16.4/34.3)	28.4 (19.3/35.4)	0.97	26.6 (18.5/37.1)	23.4 (14.5/28.8)	0.014
Muscle mass, %	32.7 (27.5/36.3)	32.0 (26.4/35.5)	0.60	30.5 (26.3/35.4)	33.5 (31.1/38.4)	0.003
Visceral fat, %	9.0 (7.0/12.0)	10.0 (7.0/14.0)	0.19	9.0 (7.0/13.0)	10 (6.0/13.2)	0.66

Baseline characteristics according to the four geriatric assessment scales—Part 2							
	EFT Score		*p* value	Fried Frailty Score			*p* value
	< 3 *n* = 32	≥ 3 *n* = 39		0 *n* = 11	1–2 *n* = 48	≥ 3 *n* = 68	
Age, years	75.7 (8.7)	76.2 (10.6)	0.85	68.8 (10.0)	75.6 (9.7)	77.0 (8.6)	0.84
Female sex, *n* (%)	16 (50)	17 (43.6)	0.59	6 (54.5)	24 (50)	33 (48.5)	0.93
BMI, kg/m^2^	27.0 (4.3)	26.7 (6.4)	0.28	25.4 (3.5)	26.0 (4.5)	27.1 (5.6)	0.85
Creatinine, mg/dl	1.1 (0.9/1.3)	1.6 (1.1/2.2)	0.73	0.9 (0.8/1.0)	1.1 (0.9/1.5)	1.4 (1.0/2.0)	0.75
Troponin T, ng/l	20.4 (9.3/24.4)	36.6 (18.4/69.4)	0.001	11.3 (4.0/19.0)	18.1 (9.8/33.2)	31.6 (18.6/59.3)	0.076
NT-proBNP, pg/ml	1451 (766/2746)	4887 (2103/14483)	0.001	264 (125/568)	1780 (911/3365)	3547 (28,450/8951)	0.30
CRP, mg/l	2.0 (0.9/4.7)	8.9 (3.8/16.6)	< 0.001	1.0 (0.6/1.9)	2.8 (1.4/8.0)	7.3 (2.8/17.3)	0.11
Hemoglobin, g/dl	14.2 (13.0/14.7)	10.7 (8.9/11.7)	< 0.001	14.0 (12.9/14.8)	12.7 (11.4/14.0)	11.3 (9.9/13.3)	0.18
Ejection fraction, %	54.7 (12.0)	47.8 (13.6)	0.03	63.7 (1.4)	50.4 (13.7)	49.7 (14.9)	0.68
sPAP, mmHg	40.4 (17.8)	44.5 (22.0)	0.28	31.0 (10.0)	37.0 (16.8)	46.9 (19.2)	0.001
EuroSCORE	11.3 (5.8/24.2)	22.0 (13.8/28.6))	0.048	4.8 (1.6/7.4)	12.5 (9.0/21.7)	18.5 (11.9/30.7)	0.12
EuroSCORE II	4.3 (2.2/6.9)	6.7 (4.0/9.9)	0.029	1.5 (0.7/1.9)	3.9 (2.4/6.3)	6.0 (3.9/9.3)	0.31
STS-PROM	2.0 (1.1/4.0)	4.3 (2.6/5.9)	0.005	0.9 (0.4/1.6)	2.7 (1.5/4.2)	3.7 (1.9/5.4)	0.089
Extracardiac arteriopathy, *n* (%)	11 (34.4)	20 (51.3)	0.15	1 (9.1)	20 (41.7)	24 (35.3)	0.12
Carotid disease, *n* (%)	8 (25)	11 (28.2)	0.76	–	15 (31.3)	11 (16.2)	0.03
COPD, *n* (%)	3 (9.4)	12 (30.8)	0.028	–	10 (20.8)	17 (25)	0.17
Smoker, *n* (%)	5 (15.6)	15 (38.5)	0.024	1 (9.1)	14 (29.2)	21 (30.8)	0.67
Hypertension, *n* (%)	25 (78.1)	34 (87.2)	0.31	6 (54.5)	35 (72.9)	58 (85.3)	0.04
Diabetes, *n* (%)	6 (18.8)	11 (28.2)	0.35	1 (9.1)	9 (18.8)	25 (36.8)	0.36
CAD, *n* (%)	16 (50)	24 (61.5)	0.33	3 (27.3)	25 (52.1)	40 (58.8)	0.14
Previous cardiac surgery, *n* (%)	9 (28.1)	9 (23.1)	0.63	1 (9.1)	8 (16.7)	20 (29.4)	0.14
Atrial fibrillation, *n* (%)	22 (68.8)	31 (79.5)	0.30	4 (36.4)	35 (72.9)	51 (75)	0.03
Body fat, %	25.4 (17.4/38.1)	22.8 (15.3/30.9)	0.58	26.4 (16.0/37.2)	24.1 (18.0/30.8)	26.8 (15.5/35.4)	0.2
Muscle mass, %	32.4 (26.6/36.7)	32.9 (30.2/36.9)	0.46	31.5 (26.4/39.1)	33.3 (28.8/35.9)	31.8 (27.5/36.5)	0.26
Visceral fat, %	11 (7.0/12.25)	10.0 (6.0/13.0)	0.66	7.0 (7.0/12.0)	10 (7.0/12.0)	10 (7.0/13.0)	0.70

Approximately, normal distributed continuous parameters were compared with Student’s *t*-test, otherwise Mann–Whitney *U* test and *χ*^2^ test were used

*CONUT Score* Controlling Nutritional Status Score, *EFT Score* Essential Frailty Toolset Score, *BMI* body mass index, *NT-proBNP* B-type natriuretic peptide, *CRP* C-reactive protein, *COPD* chronic obstructive pulmonary disease, *CAD* coronary artery disease, *MR* mitral regurgitation, *TR* tricuspid regurgitation, *sPAP* systolic pulmonary artery pressure

### Clinical outcomes according to the geriatric assessment tools

**Fig. 3:**
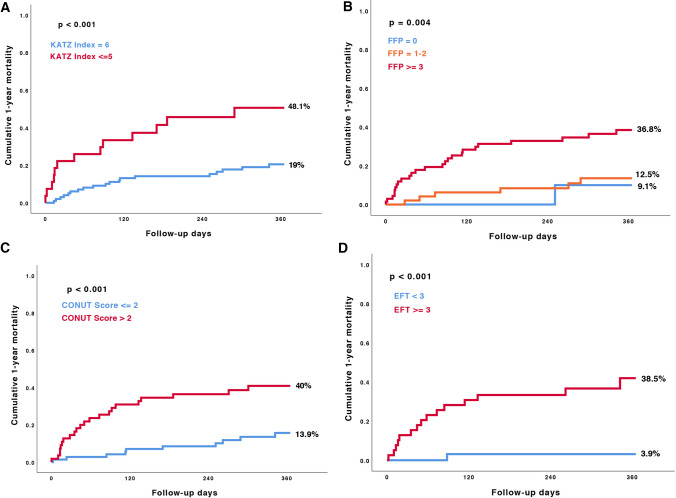
Kaplan–Meier survival analysis for one-year all-cause mortality according to the four geriatric assessment tools. *FFP* fried frailty phenotype; *CONUT Score* Controlling Nutritional Status Score; *EFT Score* Essential Frailty Toolset Score

Clinical outcomes, grouped according to the geriatric scales, are presented in Table 3. We found a significant association between frailty and geriatric syndromes and the one-year all-cause mortality rate in patients with severe MR (CONUT Score ≤ 2: 13.9% vs. CONUT Score > 2: 40%; *p* < 0.001), (Katz Index = 6: 19% vs. Katz Index ≤ 5: 48.1% *p* < 0.001), (EFT Score < 3: 3.1% vs. EFT Score ≥ 3: 38.5%; *p* < 0.001), (FFP = 0: 9.1% vs. FFP = 1–2: 12.5% vs. FFP ≥ 3: 36.8%; *p* = 0.004), as presented in Fig. 3A–D.

**Table 3:** Outcome data according to the four geriatric assessment scales

	Katz Index		*p* value	CONUT Score		*p* value
	≤ 5 *n* = 27	= 6 *n* = 100		≤ 2 *n* = 72	> 2 *n* = 55	
30-day mortality	6 (22.2)	4 (4.0)	0.002	2 (2.8)	8 (14.5)	0.015
180-day mortality	12 (44.4)	16 (16.0)	0.002	9 (12.5)	19 (34.5)	0.003
1-year mortality	13 (48.1)	19 (19.0)	< 0.001	10 (13.9)	22 (40.0)	< 0.001

	EFT score		*p* value	Fried frailty score patients with a score >= 3		*p* value
	< 3 *n* = 32	≥ 3 *n* = 39		0 *n* = 11	1–2 *n* = 48	
30-day mortality	–	5 (12.8)	0.037	–	1 (2.1)	9 (13.2)
180-day mortality	1 (3.1)	15 (38.5)	< 0.001	–	5 (10.4)	23 (33.8)
1-year mortality	1 (3.1)	15 (38.5)	< 0.001	1 (9.1)	6 (12.5)	25 (36.8)

Approximately, normal distributed continuous parameters were compared with Student’s *t*-test, otherwise Mann–Whitney *U* test and *χ*^2^ test were used

*CONUT Score* Controlling Nutritional Status Score, *EFT Score* Essential Frailty Toolset Score

### Subgroup analysis according to the treatment strategy

Out of 127 study patients, 100 underwent mitral valve repair during the follow-up period. The type of intervention is presented in Table 4. Comparing mortality rates of interventional/surgical or medical treatment strategies, we found a 5.8% absolute risk reduction in favor of a transcatheter/surgical procedure (MVR: 23.8% vs. conservative treatment: 29.6%; *p* = 0.42), which failed to reach statistical significance, presumably due to the relatively small sample size.

**Table 4:** Type of intervention

	Mitral valve repair *n* = 100
Intervention type	
MitraClip, *n* (%)	79 (79.0)
Cardioband, *n* (%)	3 (3.0)
NeoChord, *n* (%)	5 (5.0)
Valve-in-valve, *n* (%)	1 (1.0)
Mitral valve surgery, *n* (%)	11 (11.0)
CardioValve, *n* (%)	1 (1.0)

Approximately normal distributed continuous parameters were compared with Student’s t-test, otherwise Mann–Whitney *U* test and *χ*^2^ test were used

### Multivariate regression analysis

A multivariate analysis including parameters associated with mortality is presented in Table 5. The EFT Score and the FFP were independent predictors of one-year all-cause mortality (EFT Score: HR 1.9, 95% CI 1.2 to 3.2; *p* = 0.01; FFP: HR 1.8, 95% CI 1.1 to 3.1; *p* = 0.015) as well as chronic obstructive pulmonary disease (COPD) (HR 5.9, 95% CI 2.1 to 17.0; *p* < 0.001). Comorbidities such as chronic renal failure, reduced LV-EF, diabetes, or lES, ES-II and STS-PROM risk scores were not independently associated with one-year all-cause mortality.

**Table 5:** Multivariate regression analysis including the most important confounders for one-year mortality

	Univariate analysis HR (95% CI)	*p* value	Multivariate analysis HR (95% CI)	*p* value
Chronic renal failure	3.3 (1.6–6.7)	0.001	1.4 (0.2–3.3)	0.78
COPD	2.4 (1.1–4.9)	0.019	5.9 (2.1–17.0)	**0.001**
Ejection fraction	0.96 (0.94–0.98)	0.001	0.98 (0.94–1.0)	0.27
Diabetes	2.2 (1.1–4.6)	0.02	1.1 (0.2–4.4)	0.87
STS-PROM	1.1 (1.0–1.3)	<0.001	1.0 (0.8–1.1)	0.65
Logistic EuroSCORE	1.0 (1.01–1.05)	<0.001	1.0 (0.98–1.1)	0.34
EuroSCORE II	1.06 (1.02–1.1)	0.003	0.9 (0.8–1.0)	0.36
NT-proBNP	1.0 (1.0–1.0)	0.002	1.0 (1.0–1.0)	0.62
Troponin T	1.0 (0.9–1.0)	–	–	–
CONUT Score	3.5 (1.6–7.4)	0.001	1.2 (0.3–4.2)	0.75
KATZ Index	3.3 (1.6–6.7)	0.001	0.8 (0.2–4.4)	0.87
EFT Score	15.1 (2.0–114.0)	<0.001	1.9 (1.2–3.2)	**0.01**
Fried Frailty Phenotype	3.0 (1.4–6.2)	0.003	1.8 (1.1–3.1)	**0.015**

P-values marked with bold indicate statistically significant results in multivariate analysis

Approximately, normal distributed continuous parameters were compared with Student’s *t*-test, otherwise Mann–Whitney *U* test and *χ*^2^ test were used

*COPD* chronic obstructive pulmonary disease, *CONUT Score* Controlling Nutritional Status Score, *EFT Score* Essential Frailty Toolset Score

### Receiver operating characteristics curve analysis

**Fig. 4:**
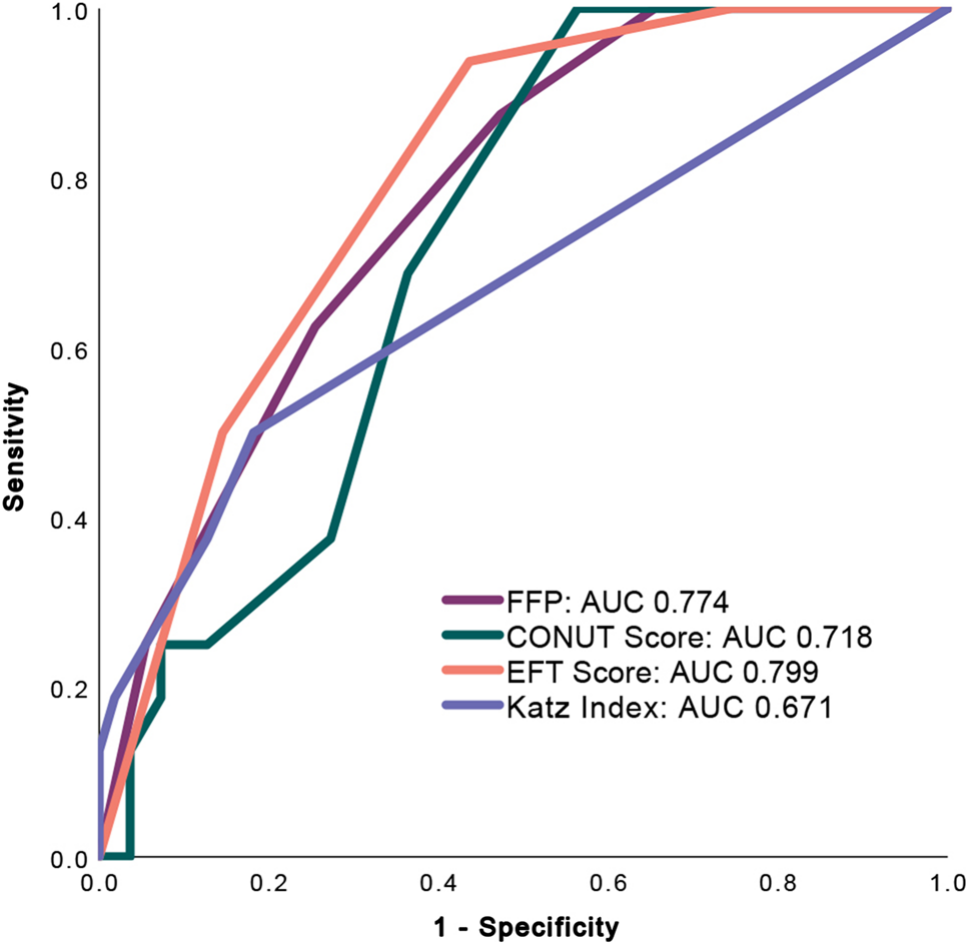
Receiver operating characteristics curve analysis comparing the predictive value of the different geriatric assessment scales for one-year all-cause mortality. *FFP* fried frailty phenotype; *CONUT Score* Controlling Nutritional Status Score; *EFT Score* Essential Frailty Toolset Score

In a receiver operating characteristics (ROC) curve analysis, comparing the predictive value of the different geriatric assessment tools for one-year all-cause mortality, the EFT Score (AUC 0.799 [95% CI 0.692–0.907], *p* < 0.001) and FFP (AUC 0.774 [95% CI 0.658–0.890], *p* = 0.001) showed the strongest association with mortality, as presented in Fig. 4.

## Discussion

The present study assessed the impact of geriatric syndromes on outcomes in older adults suffering from severe symptomatic mitral valve regurgitation and evaluated the prognostic value of different geriatric assessment tools for one-year mortality in this patient cohort.

The key findings of our study can be summarized as follows: First, geriatric syndromes are a common finding in patients with severe mitral valve regurgitation. Second, the presence of geriatric syndromes is associated with higher rates of one-year all-cause mortality and is an independent predictor for adverse outcome in patients with severe MR. Third, the EFT Score and FFP showed the strongest association with mortality and thus seem to be the most suitable assessment tools in this patient cohort.

The prevalence of geriatric syndromes in patients with severe MR varies depending on the used geriatric assessment tool and ranges from 68% assessed with the FFP to 27% using the Katz Index in our study cohort. While some previous studies showed a relatively low prevalence of geriatric syndromes in patients undergoing transcatheter mitral valve repair (TMVR) or TAVR (TMVR 9.8% and TAVR 8.1%) [13], other investigators found that approximately half of the patients (45.5%) undergoing TMVR suffer from frailty [14]. A similar prevalence of frailty has been described in patients with severe aortic stenosis (AS) undergoing transcatheter aortic valve replacement [4], whereas the incidence of frailty is twofold lower in patients with AS undergoing surgical aortic valve replacement (SAVR) [3]. These inconsistencies may be explained by the use of different frailty assessment tools and cut-off values, since standardized frailty scores are—in contrast to surgical risk scores—not yet implemented in the European guidelines or Heart Team risk assessment [15].

Geriatric assessment could play a pivotal role in patient selection given the negative effect of frailty on outcome in patients with cardiovascular disease [16] and heart failure [17, 18]. Especially, in patients undergoing TAVR, who usually represent an older and multimorbid patient population, frailty acts as a risk factor for death and disability [3] and independently predicts one-year mortality (OR 2.75, 95% CI 1.55–4.87, *p* < 0.001) [4). Similar results have been reported in patients undergoing TMVR by Metze et al. [14]. The authors described significantly higher hazards of death (hazard ratio: 3.06; *p* = 0.001) and death or heart failure decompensation (hazard ratio: 2.03; *p* = 0.007) in frail patients with severe MR undergoing TMVR compared to non-frail patients. Miura et al*.* [19] found that malnutrition and frailty were predictors for adverse 6-month outcomes in hospitalized patients with heart failure and untreated mitral valve regurgitation and in patients with chronic heart failure (HF), while Yang et al*.* [18] found 1.5-fold higher rates of mortality and incident hospitalization according to the frailty status. However, these results are not surprising, since HF and frailty share common clinical signs and symptoms, such as sarcopenia, cachexia, muscle weakness, reduced cardiac fitness, and increased inflammatory potential [18].

In our study, frailty and geriatric syndromes, as assessed with four different scales, were significantly associated with higher rates of one-year all-cause mortality in patients with severe MR, who had already undergone transcatheter or surgical valve therapies or were scheduled for TVT or treated conservatively. Furthermore, we found an accumulation of concomitant comorbidities that was also reflected in higher surgical risk scores in frail patients, representing a higher morbidity burden and emphasizing that these patients suffer from a more advanced disease state. Our findings suggest that geriatric syndromes are important predictors for outcomes in patients with severe regurgitation of the mitral valve and that its assessment could and should be part of the Heart Team discussion.

In spite of these conclusions, the standardized assessment of frailty and geriatric syndromes remains challenging. Around 35 different scales to assess frailty exist [20] and additional items such as malnutrition and functional impairments are used to evaluate other geriatric syndromes. In our study, we compared the incremental predictive value of four geriatric scales on the outcome: the CONUT Score, the Katz Index, the FFP, and the EFT Score. In a multivariate regression analysis, the EFT Score and FFP were independent predictors for one-year all-cause mortality even after adjusting for surgical risk scores and the NT-pro BNP levels, which were only associated with the outcome in univariate analyses. This result is surprising, since the surgical risk scores and the NT-pro BNP levels represent important markers of heart failure and have been shown to be independently associated with outcome in patients undergoing TAVR [21, 22], especially for heart failure patients [23]. In patients with severe MR, however, validated data for risk assessment before transcatheter or surgical procedures are not available to the best of our knowledge [22, 24]. Previous studies investigating the applicability of surgical risk scores in patients undergoing TMVR showed that the lES, ES-II, and STS-PROM overestimated the risk of mortality at 30 days and were incorrectly calibrated at 2 and 3 years. Only at 1 year, there was a good alignment between the observed and predicted probabilities for ES-II and STS-PROM, whereas lES overestimated the mortality risk [24] demonstrating the need for better risk assessment tools in patients with severe mitral valve regurgitation.

In our study population, the FFP and EFT were independent predictors for one-year all-cause mortality. However, apart from a strong predictive reliability, ease of use is important for daily practice. The FFP combines functional performance tests, such as the evaluation of grip strength and walking time, with physical examinations including the level of physical activity, exhaustion in daily life, and weight loss, whereas the EFT Score which combines functional, cognitive and laboratory parameters seems to be more feasible in a routine clinical setting. In addition, interobserver reliability appears to be superior [25]. Another advantage over the FFP is that the EFT Score does not require specialized equipment.

Altogether, the EFT Score may be a suitable geriatric assessment tool to predict mortality in older adults with severe mitral valve regurgitation. The question of whether patients with advanced geriatric syndromes and frailty should be treated preferably conservatively or whether this patient cohort especially profits from mitral valve repair remains unanswered. Larger trials are necessary to elucidate this important question.

## Study limitations

Two important limitations of our study are the small sample size and its monocentric character. Furthermore, the median follow-up duration of 343 days provides insights into the impact of geriatric syndromes in the mid-term but precludes extrapolating the results to a longer-term follow-up. To address selection and therapy bias, we performed a subgroup analysis comparing rates of mortality according to the different treatment strategies. However, a certain selection bias may prevail since only high-risk patients were included.

Our results are hypothesis generating. Further prospective and randomized trials are needed to confirm the results.

## Conclusion

Geriatric syndromes are associated with mortality in patients with severe MR. The EFT Score and FFP were independent predictors of one-year all-cause mortality and seem to be the most suitable assessment tools to predict mortality in older adults with severe MR.

## Abbreviations

MR: Mitral valve regurgitation
TAVR: Transcatheter aortic valve replacement
CONUT Score: Controlling Nutritional Status Score
EFT Score: Essential Frailty Toolset Score
FFP: Fried frailty phenotype
MVR: Mitral valve repair
NT-proBNP: B-type natriuretic peptide
COPD: Chronic obstructive pulmonary disease
CAD: Coronary artery disease
